# A novel qualitative signature based on lncRNA pairs for prognosis prediction in hepatocellular carcinoma

**DOI:** 10.1186/s12935-022-02507-z

**Published:** 2022-02-22

**Authors:** Xiaoyun Bu, Luyao Ma, Shuang Liu, Dongsheng Wen, Anna Kan, Yujie Xu, Xuanjia Lin, Ming Shi

**Affiliations:** 1 Department of Liver Surgery, Sun Yat-Sen University Cancer Center, Guangzhou, China; 2 State Key Laboratory of Oncology in South China, Collaborative Innovation Center for Cancer Medicine, 651 Dongfeng East Road, Guangzhou, 510060 China; 3 Guizhou Medical University, Guiyang, China; 4 Department of Hepatic-Biliary-Pancreatic Surgery, The Affiliated Hospital of Guizhou Medical University, Guiyang, China; 5 Key Laboratory of Hepatobiliary and Pancreatic Surgery, Guiyang, China

**Keywords:** Hepatocellular carcinoma, LncRNA pairs, Risk stratification, Precision medicine

## Abstract

**Background:**

Prognostic assessment is imperative for clinical management of patients with hepatocellular carcinoma (HCC). Most reported prognostic signatures are based on risk scores summarized from quantitative expression level of candidate genes, which are vulnerable against experimental batch effects and impractical for clinical application. We aimed to develop a robust qualitative signature to assess individual survival risk for HCC patients.

**Methods:**

Long non-coding RNA (lncRNA) pairs correlated with overall survival (OS) were identified and an optimal combination of lncRNA pairs based on the majority voting rule was selected as a classification signature to predict the overall survival risk in the cancer genome atlas (TCGA). Then, the signature was further validated in two external datasets. Besides, biomolecular characteristics, immune infiltration status, and chemotherapeutics efficacy of different risk groups were further compared. Finally, we performed key lncRNA screening and validated it in vitro.

**Results:**

A signature consisting of 50 lncRNA pairs (50-LPS) was identified in TCGA and successfully validated in external datasets. Patients in the high-risk group, when at least 25 of the 50-LPS voted for high risk, had significantly worse OS than the low-risk group. Multivariate Cox, receiver operating characteristic (ROC) curve and decision curve analyses (DCA) demonstrated that the 50-LPS was an independent prognostic factor and more powerful than other available clinical factors in OS prediction. Comparison analyses indicated that different risk groups had distinct biomolecular characteristics, immune infiltration status, and chemotherapeutics efficacy. TDRKH-AS1 was confirmed as a key lncRNA and associated with cell growth of HCC.

**Conclusions:**

The 50-LPS could not only predict the prognosis of HCC patients robustly and individually, but also provide theoretical basis for therapy. Besides, TDRKH-AS1 was identified as a key lncRNA in the proliferation of HCC. The 50-LPS might guide personalized therapy for HCC patients in clinical practice.

**Supplementary Information:**

The online version contains supplementary material available at 10.1186/s12935-022-02507-z.

## Background

Liver cancer is one of the most lethal cancers worldwide and approximately 75–85% of the cases are diagnosed as hepatocellular carcinoma (HCC) pathologically [1]. For HCC patients with proper preserved liver function, curative surgical resection still remains the major treatment [2]. However, the prognosis of HCC patients varied tremendously after surgery due to tumor heterogeneity [2, 3], highlighting the need for personalized managements. Therefore, it is of critical importance to predict the prognosis of HCC patients after surgery for guiding clinical therapy and patient management.

Currently used clinical features and biomarkers, such as TNM staging system, BCLC staging system, and serum alpha-fetoprotein (AFP) level, were insufficient in providing accurate prognostic evaluation for HCC patients in clinical practice [4, 5]. For example, the most widely used serum AFP level was reported to be nearly half-negative in early and small size HCC patients [6], which crippled the prognostic ability of serum AFP. Moreover, the TNM and BCLC staging system were not widely used for the differences in etiology and genetic background of HCC patients [4].

Although many prognostic signatures based on scoring from quantitative gene expression have been developed for predicting prognosis of HCC patients recently [7–9], most of them require pre-collection of samples for data normalization and have inconsistent cut-off value in different cohorts, which are impractical for clinical application. Conversely, the within-sample relative expression orderings (REOs) of gene pairs, which is an individual qualitative transcriptional trait, has been demonstrated to be robust against batch effects and data normalization [10, 11]. Based on the REOs, some qualitative signatures were created and achieved satisfactory performance in prognosis prediction for several cancers [12–15]. However, most reported qualitative prognostic signatures were based on protein-coding genes and few of them focused on HCC. Considering the important role of long noncoding RNA (lncRNA) in the carcinogenesis of HCC [16], it is worthy to find an individualized lncRNA prognostic signature for HCC patients after surgery.

In this study, a novel REOs-based prognostic signature consisting of 50 lncRNA pairs (50-LPS) was identified in a training dataset, and the signature was successfully validated in two external datasets. We further revealed the different biomolecular characteristics, immune infiltration status, and chemotherapeutics efficacy between the two prognostic groups. Moreover, among the 55 lncRNAs included in the 50-LPS, TDRKH-AS1 was confirmed as a key lncRNA and associated with cell growth of HCC. In clinical practice, these results might be useful for aiding personalized therapy and management of HCC patients.

## Methods

### Data source and preprocessing

All the cohorts of HCC samples, as described in Additional file 1: Table S1, were downloaded from public resources. Gene expression profile and somatic mutation data of the TCGA-LIHC cohort were obtained by using R package *TCGAbiolinks* [17] and *TCGAmutations* [18] respectively. Clinical information of the TCGA-LIHC cohort was downloaded from UCSC Xeno (https://xena.ucsc.edu/public) on 2019-11-10. Transcriptome data of CHCC cohort was accessed from NODE (https://www.biosino.org/node) and the corresponding clinical information was acquired from a previous study [19]. Transcriptome and clinical information of LIRI samples, included in the PCAWG project, were downloaded from ICGC (https://dcc.icgc.org/releases/PCAWG) [20]. GSE77509 and GSE104580 were downloaded from the Gene Expression Omnibus (GEO) database. After mapping the ensemble gene IDs to gene names according to the annotation files (hg38.99) downloaded from Ensemble (http://asia.ensembl.org), all the gene expression values were transformed into transcripts per million (TPM) values. LncRNAs were kept for further analysis, whose expression value was more than zero in more than 90% of samples both in TCGA-LIHC and CHCC cohorts.

### Survival analysis

The overall survival (OS) interval was defined as the time from surgery to death or the last follow-up. Kaplan–Meier survival curves and log-rank test were used to evaluate the difference of OS between the high-risk and low-risk subgroups. The univariate Cox regression model was utilized to identify prognostic lncRNA pairs and clinical factors for HCC patients. The multivariate Cox regression model was used to evaluate prognostic performance of the signature after adjusting for age, gender, serum AFP levels, liver cirrhosis, tumor vascular invasion (VI), and TNM stage. Hazard ratios (HRs) and 95% confidence intervals (CIs) were calculated from the Cox proportional- hazards model. The predictive ability of the signature was evaluated by the concordance index (C-index) value and the AUC value of the ROC curve [21]. Decision curve analysis (DCA) was used to assess the utility of the signature for decision making [22].

### Identification of the signature

First, lncRNAs with significant prognostic value were identified by using univariate Cox regression analyses in the training dataset (TCGA-LIHC). Then, lncRNA pairs were constructed via permutation and combination by R software. For a given lncRNA pair, for example, lncRNA 1 and lncRNA 2 with expression levels of E1 and E2, its REO pattern (E1 > E2 or E1 < E2) can stratify all samples into two groups. If the two groups have significantly different OS, the lncRNA pair is considered as a candidate prognostic lncRNA pair. Next, a forward selection procedure was adopted to seek an optimal subset of the lncRNA pairs, which was based on the pre-defined classification rule: a patient is classified into the high-risk group if no less than half of the lncRNA pairs of this patient vote for high risk; otherwise, the low-risk group. In brief, the candidate lncRNA pair with the highest C-index was selected as the seed signature, and the other prognosis‐related lncRNA pairs, ranked in descending C-index order, were added into the signature one by one. Every time an additional lncRNA pair added in the signature, a new C-index value was calculated to check if the predictive ability of the signature was better than before. Finally, the optimal REO lncRNA signature was identified when an addition of a new lncRNA pair did not improve the C-index of the signature in the training dataset.

### Analyses of transcriptional and genomic data

The Wilcoxon rank-sum test was performed to identify differentially expressed genes between the low- and high-risk subgroups. Gene Set Enrichment Analysis (GSEA) was conducted by using *clusterProfiler* R package [23] to explore the difference of biological characteristics between the two groups. Genes with different mutation frequencies between the two prognostic groups were visualized by *complexheatmap* R package [24].

### Estimation of immune infiltration

CIBERSORT algorithm [25] was adopted to investigate the immune-cell infiltration. The differences of immune infiltrating cell scores were analyzed by Wilcoxon rank-sum test.

### Characterization of HCC subclasses

To analyze the correlation between the two prognostic subgroups and previously published HCC molecular subtypes, MS.liverK algorithm [26] was used to characterize the six different molecular subtypes in TCGA-LIHC samples. Chi-square test was used to detect correlations between the two subgroups and HCC subclasses.

### Prediction of the benefit of each risk group from chemotherapy

Our previous data (GSE104580) consisting of HCC patients treated with transarterial chemoembolization (TACE) was used to indirectly predict the chemotherapy efficacy of the two risk subgroups based on subclass mapping analysis [27]. In addition, to test whether the subgroup could benefit from other chemotherapeutic drugs, we downloaded the predicted half maximal inhibitory concentration (IC50) data of TCGA-LIHC samples from a previous study [28]. Wilcoxon rank-sum test was used to detect the difference of predicted IC50 value between the two groups.

### Hub lncRNAs screening and validation in vitro

Hub lncRNAs were defined as the differentially expressed lncRNAs with log_2_(fold change) > 1 and FDR < 0.05 (Wilcoxon test) between tumor and normal liver tissues, as well as associated with OS in TCGA-LIHC cohort and CHCC cohort. To validate the expression pattern between tumor and normal liver tissues by quantitative real‑time PCR, we collected five paired HCC and normal liver tissues in our center. This research was approved by the Ethics Committee of Sun Yat-Sen University Cancer Center, and written informed consent was obtained from all patients. In addition, we conducted in vitro assays to investigate the biological function of the key lncRNA.

### RNA extraction, reverse transcription, and quantitative real‑time PCR

According to the manufacturer’s instructions, total RNA was isolated by using an RNA Extraction Kit (ESscience Biotech, Shanghai, China). For reverse transcription, 2 μg of total RNA was used to synthesize cDNA with a cDNA Synthesis Kit (TOYOBO, Osaka, Japan). Then cDNA was subjected to quantitative real‑time PCR amplification using SYBR Green (TOYOBO, Osaka, Japan) with a Bio-Rad PCR System. β-actin was used as an internal control. The sequence of primers used in this study was provided in Additional file 1: Table S2.

### Cell lines and culture conditions

HCC cell lines, MHCC97H and Huh7, were obtained from Shanghai Municipal Liver Cancer Medical Center and the National Collection of Authenticated Cell Cultures, respectively. The cell lines were incubated in Dulbecco’s modified Eagle’s medium (Gibco, Carlsbad, USA) with 10% foetal bovine serum (Gibco, Carlsbad, USA), with 5% CO_2_ at 37 °C.

### Plasmid construction and transfection

The pSLenti vector containing short hairpin RNAs (shRNA) targeting TDRKH-AS1 was obtained from OBiO Technology (OBiO, Shanghai, China). According to the instructions, plasmid was transfected into MHCC97H and Huh7 cell line using a Lenti-Pac™ HIV Expression Packaging Kit (GeneCopoeia, Rockville, USA). The sequence of the shRNA was provided in Additional file 1: Table S2.

### In vitro cell growth assays and apoptosis assays

For CCK8 assays, the transfected cells were plated in 96-well plates at a density of 2000 cells per well. Optical Density (OD) value at 450 nm was tested on day 0, 1, 2, 3, and 4 with a CCK8 assay kit (Dojindo, Kumamoto, Japan). For Cell colony formation assays, the transfected cells were plated in 6-well plates at a density of 1000 cells per well and harvested on day 14. Crystal violet staining solution (Beyotime, Shanghai, China) was used to stain the cell colony. The total area of the colony was detected by Image J software (NIH Image, Bethesda, MD). According to the manufacturers’ instructions, apoptosis assays were performed by PI/Annexin V detection kits (ESscience Biotech, Shanghai, China) and analyzed by flow cytometry (Beckman CytoFLEX, USA).

### Western blotting

Cell extracts were harvested and were resolved by SDS-PAGE and transferred to a polyvinylidene difluoride membrane. After blocking, the membrane was incubated with primary antibodies including Caspase3 (1:1000, Cell Signaling Technology, USA), phospho-Akt (Ser473)(1:1000, Cell Signaling Technology, USA), GAPDH (1:1000, Cell Signaling Technology, USA) and the secondary antibody HRP-conjugated goat anti-rabbit IgG (1:3000; Cell Signaling Technology, USA). Signals were detected by an ECL kit (Bio-Rad, USA).

### Statistical analysis

All the computational and statistical analyses were performed using R 4.0.1 or GraphPad Prism 8.0.1 software. P-value < 0.05 was regarded as statistically significant. The false discovery rate (FDR) was calculated using the Benjamini–Hochberg method [29] when multiple testing occurred.

## Results

### Development of the REOs-based prognostic signature

**Fig. 1:**
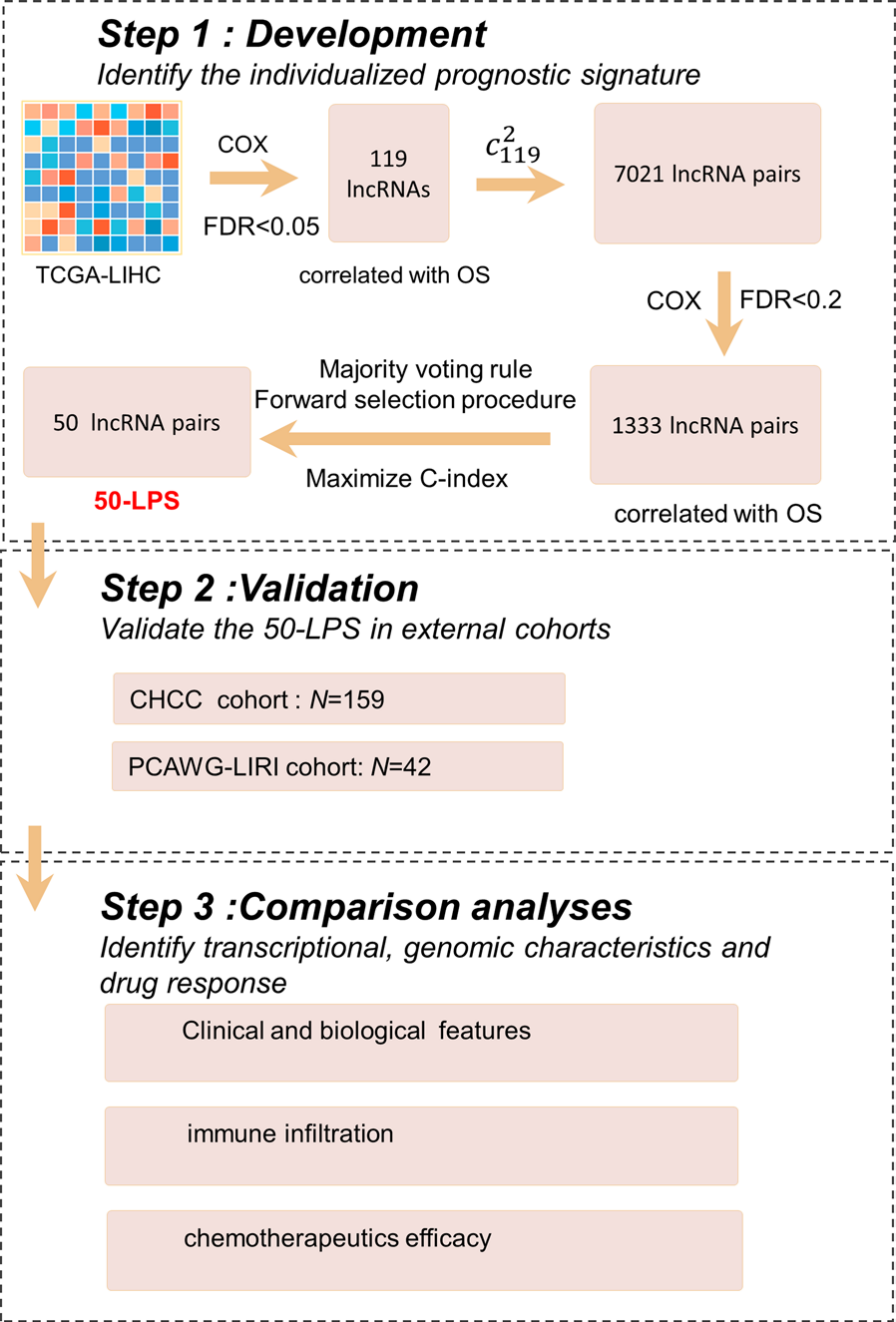
Overview of the study workflow

The main research steps of this study are summarized in Fig. 1. TCGA-LIHC cohort with 365 HCC samples was used as the training dataset. By setting a cut-off less than 0.05 of FDR in the univariate Cox proportional hazards regression model, we identified 119 OS-associated lncRNAs. For all the lncRNA pairs consisting of every two of the 119 pre-selected lncRNAs, 1333 prognosis‐associated lncRNA pairs were chosen (FDR < 0.2) and then ranked in descending order according to their C-index values. According to the majority voting rule (see “Methods” section), a final set of 50 lncRNA pairs (Table 1) attained the highest C-index value of 0.75 was identified through the forward selection procedure (see “Methods” section).

**Table 1:** The composition of the 50‐LPS

Pair 1–25	LncRNA 1	LncRNA 2	Pair 26–50	LncRNA1	LncRNA2	
Pair1	TMEM220-AS1	NRAV	Pair26	LINC01554	AL139423.1	
Pair2	TMEM220-AS1	SREBF2-AS1	Pair27	AC026362.1	LINC02487	
Pair3	LINC00324	AC074117.1	Pair28	TMEM220-AS1	ZNF433-AS1	
Pair4	LINC00324	PCAT6	Pair29	ZNF433-AS1	MAFG-DT	
Pair5	LINC01554	AC009005.1	Pair30	LINC00324	HMGN3-AS1	
Pair6	LINC00324	NCK1-DT	Pair31	PXN-AS1	CYTOR	
Pair7	AC099329.2	C2orf27A	Pair32	TMEM220-AS1	AL671710.1	
Pair8	LINC00324	LINC00205	Pair33	AC099329.2	NRAV	
Pair9	AL049840.6	DANCR	Pair34	TMEM220-AS1	LINC01011	
Pair10	LINC02362	TDRKH-AS1	Pair35	LINC00324	MAPKAPK5-AS1	
Pair11	GHRLOS	TMCC1-AS1	Pair36	LINC00324	SLC16A1-AS1	
Pair12	TMEM220-AS1	MKLN1-AS	Pair37	LINC00324	AC009403.1	
Pair13	AC099329.2	AL365203.2	Pair38	TMEM220-AS1	AL355987.4	
Pair14	ZNF337-AS1	AC012073.1	Pair39	ZNF337-AS1	GIHCG	
Pair15	AC099329.2	PCAT6	Pair40	AL359643.3	TMCC1-AS1	
Pair16	TMEM220-AS1	AP001469.3	Pair41	GHRLOS	AC027097.1	
Pair17	AC019080.1	AC124798.1	Pair42	AL050341.2	MKLN1-AS	
Pair18	PXN-AS1	AC009005.1	Pair43	AL353708.1	AL365203.2	
Pair19	AC099329.2	PXN-AS1	Pair44	NCK1-DT	LINC00513	
Pair20	TMEM220-AS1	LINC01134	Pair45	ZNF337-AS1	AL365203.2	
Pair21	NDUFB2-AS1	AC145343.1	Pair46	AC012146.1	AL355987.4	
Pair22	LINC02499	AC099850.2	Pair47	TBC1D8-AS1	AC027097.1	
Pair23	AC019080.1	LINC01134	Pair48	LINC00324	LINC01134	
Pair24	AL359643.3	AC107021.2	Pair49	GHRLOS	FOXD2-AS1	
Pair25	AC099329.2	DCST1-AS1	Pair50	PXN-AS1	GHRLOS	

**Fig. 2:**
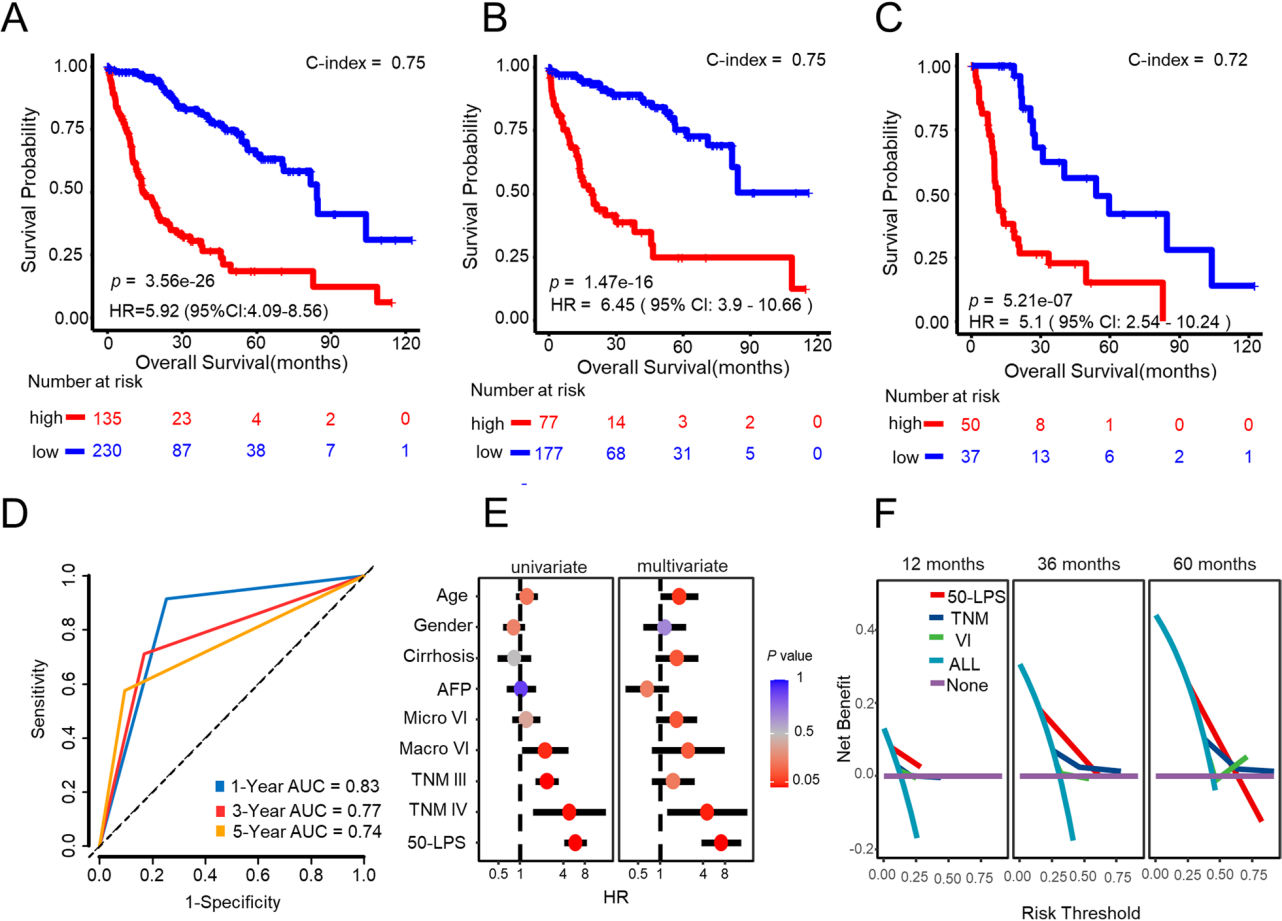
The overall survival outcome of the two risk subgroups stratified by the 50-LPS in the training cohort (TCGA-LIHC). The Kaplan–Meier curves of overall survival for the entire training cohort (**A**) and patients in TNM I + II (**B**) and TNM III + IV (**C**). The time-dependent ROC analysis of the 50-LPS in the training cohort (**D**). Univariate and multivariate Cox regression analyses of OS in the training cohort (**E**). DCA of prognostic factors identified from univariate cox in training cohort (**F**)

Using the 50 lncRNA pairs signature, named as 50-LPS, in the training cohort, 135 samples were stratified into high-risk group, who got no less than 25 lncRNA pairs voting for high risk, and other samples were stratified into low-risk group. KM plot showed that samples in the high-risk group had an obviously worse overall survival than those in the low-risk group (HR = 5.92, 95% CI 4.09–8.56, *p* = 3.56 × 10^–26^, Fig. 2A). ROC curve indicated that the 50-LPS had a robust predictive ability with an AUC value of 0.83, 0.77, 0.74 in the 1st year, 3rd year and 5th year (see Fig. 2D), respectively. What’s more, the 50-LPS maintained a significant prognostic power after adjusting for age, gender, liver cirrhosis, serum AFP level, tumor vascular invasion (VI) and TNM stage (Fig. 2E) and presented a better utility than TNM stage and VI in DCA (Fig. 2F). Peculiarly, the 50-LPS can successfully stratify the early-stage (stage I + II) samples into high- and low-risk group with significant OS difference (HR = 6.45, 95% CI 3.90–10.66, *p* = 1.47 × 10^–16^, Fig. 2B) as well as the late-stage (stage III + IV) samples (HR = 5.1, 95% CI 2.54–10.24, *p* = 5.21 × 10^–7^, Fig. 2C).

### Validation of the signature

**Fig. 3:**
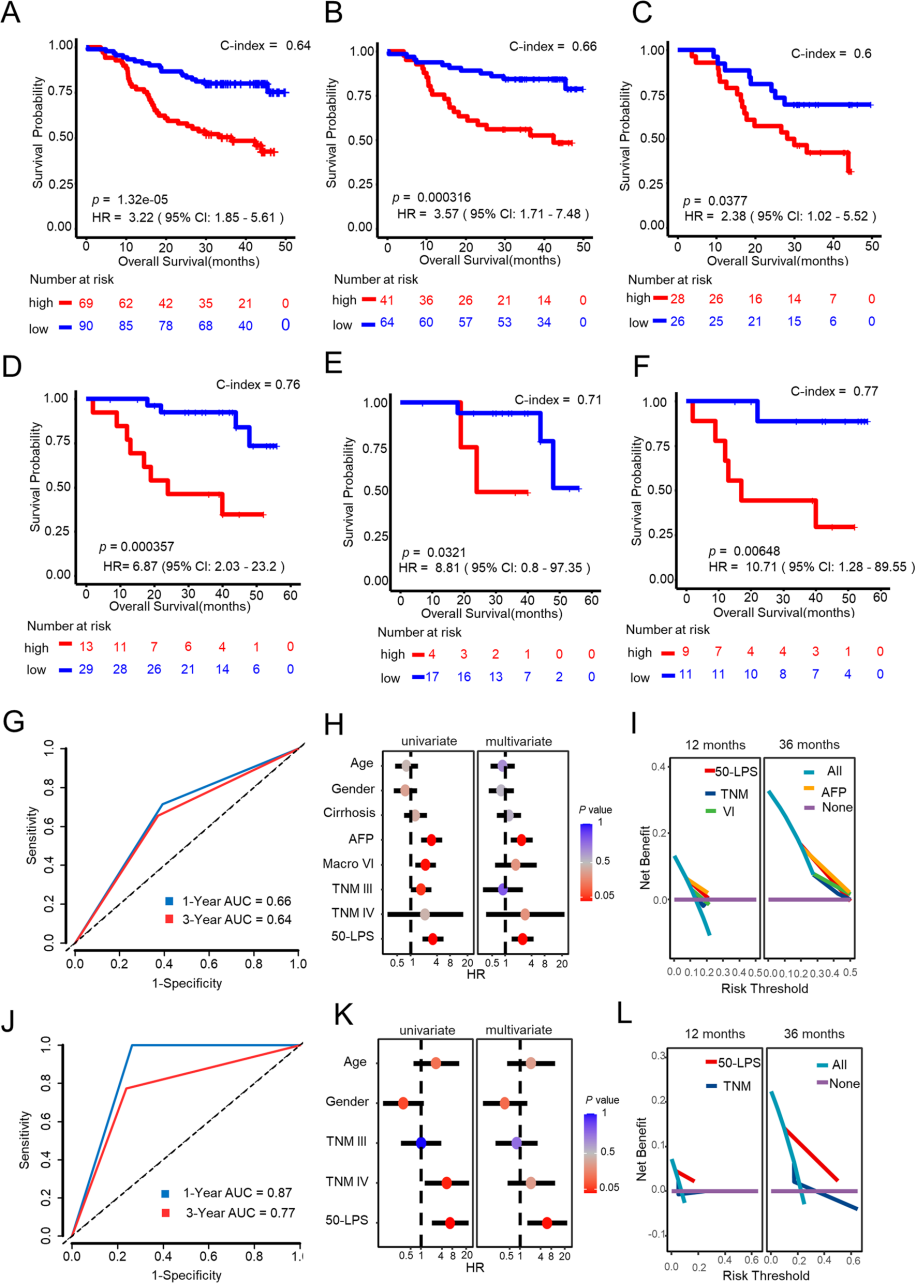
Performance of the 50-LPS in two external cohorts. The Kaplan–Meier curves of overall survival for the entire CHCC cohort (**A**) and TNM I + II (**B**) and TNM III + IV (**C**) patients. The Kaplan–Meier curves of overall survival for the entire LIRI cohort (**D**) and TNM I + II (**E**) and TNM III + IV (**F**) patients. The time-dependent ROC analysis of the 50-LPS in the CHCC cohort (**G**) and LIRI cohort (**J**). Univariate and multivariate Cox regression analyses of OS in the CHCC cohort (**H**) and LIRI cohort (**K**). DCA of prognostic factors identified from univariate cox in CHCC cohort (**I**) and LIRI cohort (**L**)

Two external HCC cohorts were used to test the predictive ability and utility of the 50-LPS. A number of 159 HBV associated HCC samples in CHCC cohort were firstly subjected to risk assessment by using the 50-LPS. 69 and 90 samples were classified into the high- and low-risk groups, respectively. The high-risk group had a significantly shorter OS when compared to the low-risk group (HR = 3.22, 95% CI 1.85–5.61, *p* = 1.32 × 10^–5^, Fig. 3A) with an AUC value of 0.66 and 0.64 at 1st year and 3rd year respectively (Fig. 3G). The 42 samples from LIRI cohort were used as the second validation set. 13 and 29 samples were predicted to be in the high- and low-risk groups with extremely different OS (HR = 6.87, 95% CI 2.03–23.2, *p* = 3.57 × 10^–4^, Fig. 3D) with an AUC value of 0.87 and 0.77 at 1st year and 3rd year respectively (Fig. 3J). In addition, the 50-LPS also succeeded in stratifying the early-stage or late-stage HCC patients from CHCC cohort (Fig. 3B, C) and LIRI cohort (Fig. 3E, F).

Furthermore, the multivariate Cox regression analysis, ROC curve and DCA were also performed in the two validation cohorts. As expected, we found that the 50-LPS exhibited a consistent powerful ability and utility for OS-prediction and dwarfed other clinical factors including TNM stage, serum AFP level, and VI (Fig. 3H, I, K, L).

### Distinct transcriptional and genomic characteristics of the two prognostic groups

**Fig. 4:**
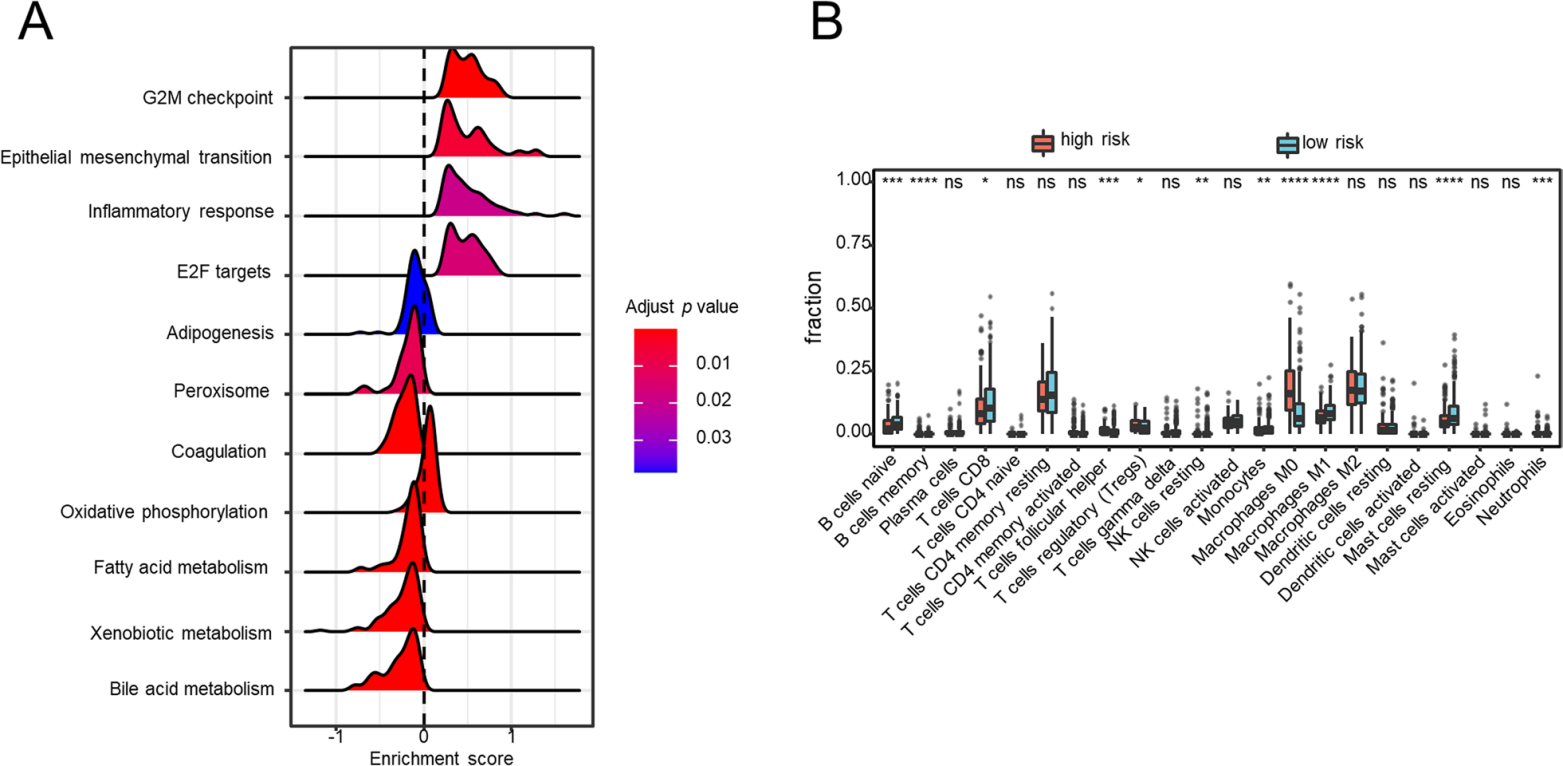
Biological features and immune cell infiltration of the two risk subgroups stratified by the 50-LPS. Pathway enrichment analysis by GSEA (**A**). The difference of immune cell infiltrated in the tumor microenvironment between the two groups (**B**). *p < 0.05, **p < 0.01, ***p < 0.001, ****p < 0.0001

In order to better characterize the biomolecular characteristics of the two risk-groups, differential analyses of their transcriptome and somatic mutation data were conducted. We obtained the differential expressed genes between the two prognostic groups and performed GSEA to identify pathways enriched in each subgroup. As shown in Fig. 4A, the high-risk group typically enriched in some pathways relevant to tumor proliferation and metastasis such as G2M checkpoint, E2F targets, epithelial-mesenchymal transition (EMT), while the low-risk group mainly enriched in several metabolism-relevant pathways including fatty acid metabolism and bile acid metabolism.

**Fig. 5:**
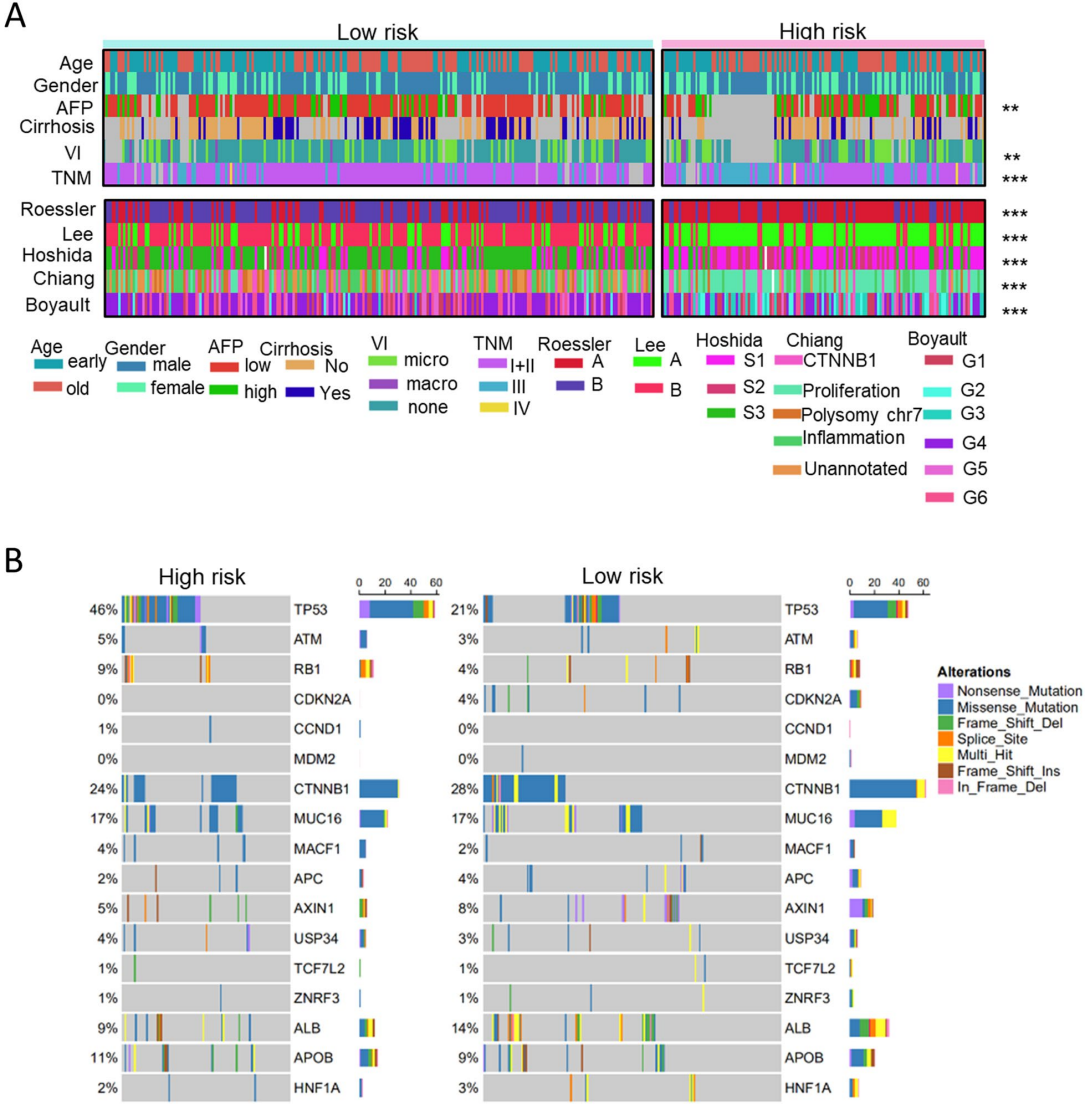
Clinical, molecular and genomic characteristics of the two risk subgroups stratified by the 50-LPS. Clinical characteristics of HCC subgroups and association with previous HCC molecular subtypes (**A**). The difference of frequencies in gene mutation between the two groups (**B**). *p < 0.05, **p < 0.01, ***p < 0.001

To further investigate the differences in somatic mutation frequency between the two subgroups, we analyzed genes with high mutation frequency or in important pathways relevant to HCC, including P53, cell cycle pathway, WNT beta-catenin pathway, and hepatic differentiation. Results in Fig. 5B showed that the high-risk group displayed a higher mutation frequency of TP53 (46%) than the low-risk group (21%), implying the dysregulation of cell proliferation in high-risk group.

### Difference of immune infiltration landscape in two HCC subgroups

Because recent study indicated that immunologic background may influence the prognostic result of HCC patients, the difference of the immune cell infiltration between the two prognostic groups deserves further elaboration. Using the CIBERSORT algorithm, we revealed that the low-risk group had a relatively higher level of tumor-infiltrating immune cells including naïve B cells, CD8^+^ T cells, monocytes, M1 macrophages and resting Mast cells, while the high-risk group had a richer infiltration of follicular helper cells, regulatory T cells, M0 macrophages and neutrophils (Fig. 4B).

### Correlation of the HCC subgroups with clinical characteristics and previously reported subclasses

We explored the relationship between the clinicopathological features and HCC subgroups classified by our novel signature in the TCGA-LIHC cohort. As illustrated in the heatmap (Fig. 5A), samples in the two subgroups had several different clinicopathological characteristics. According to the result of chi-square test, the high-risk group was correlated with advanced pathologic stage (*p* < 0.001), high serum AFP level (*p* = 0.003), and more frequent VI situation (*p* = 0.002), but displayed no difference with the low-risk group in age, gender and liver cirrhosis.

What’s more, we compared our classification with previously published HCC molecular subclasses to better understand the molecular background underlying the two prognostic subgroups. The heatmap (Fig. 5A) revealed that the high-risk group was obviously linked to Hoshida’s [30] S1/S2 subclass (*p* < 0.001), Lee’s [31] A subclass (*p* < 0.001), Roessler’s [32] subgroup A(*p* < 0.001), Chiang’s [33] proliferation (*p* < 0.001) class and Boyault’s [34] G2/G3 (*p* < 0.001), whereas the low-risk group was significantly correlated with Hoshida’s S3 (p < 0.001), Lee’s B subtype (*p* < 0.001), Chiang’s CTNNB1 (*p* < 0.001) class and Boyault’s G4/G5 subclass(*p* < 0.001).

### Correlation between the signature and chemotherapeutics

Since the two groups of patients with different OS outcomes have different biological characteristics, we then wanted to find out whether there was a difference in chemotherapeutic drug sensitivity between them.

**Fig. 6:**
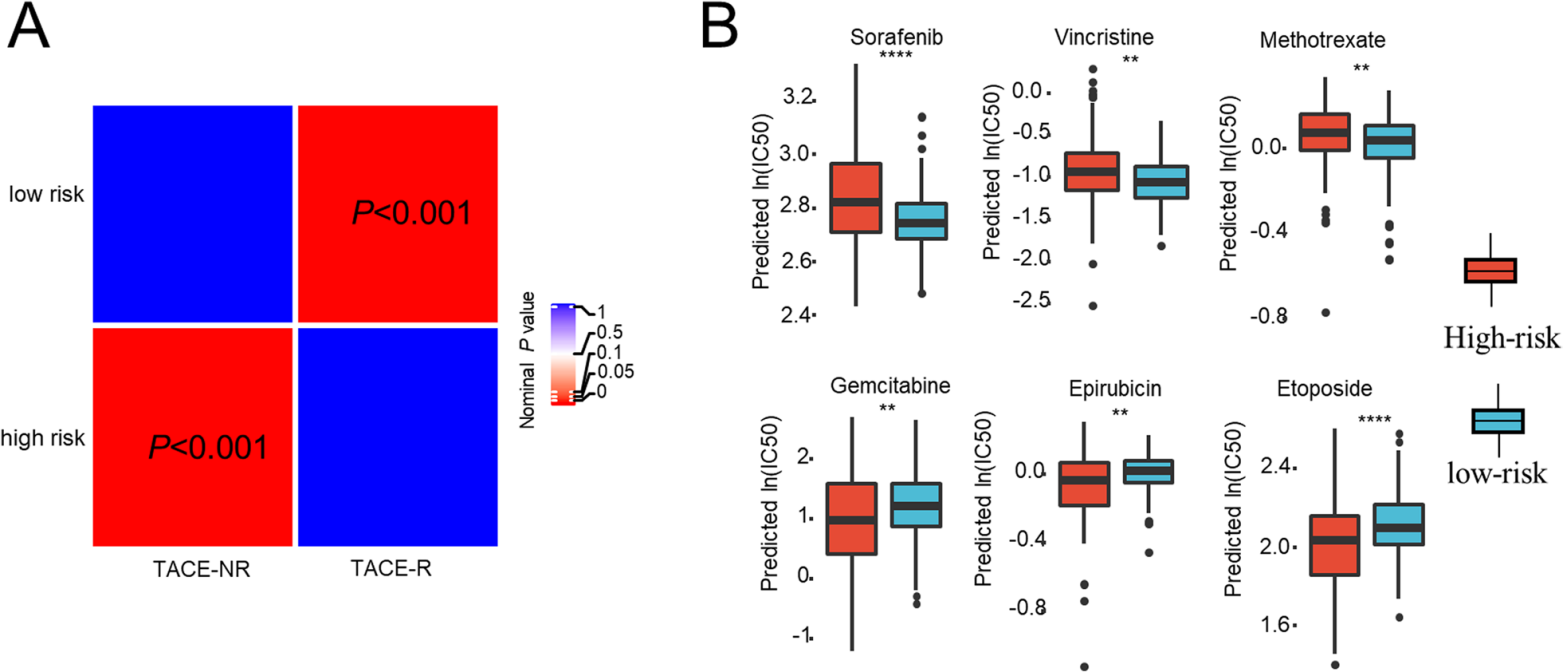
Correlations of the HCC subgroups with the response to different chemotherapeutics. Subclass mapping correlation analysis between HCC subgroups and samples with different sensitivities to TACE (**A**), and several anticancer drugs (**B**). TACE-NR, TACE non-responder; TACE-R, TACE responder. ** *p*<0.01,*** *p*<0.001,**** *p*<0.0001

Using the subclass mapping method, we compared the transcriptome profile of the two subgroups with our previous cohort containing 147 HCC patients who received TACE. Surprisingly, we found that the low-risk group was significantly correlated with the TACE-response group (*p* < 0.001, Fig. 6A), while the high-risk group showed an obvious similarity to the TACE-resistant group (*p* < 0.001, Fig. 6A), which indicated that the low-risk group but not the high-risk group would benefit from TACE therapy.

In addition, we also explored the efficacy of different antitumor agents between the two groups. The result of Wilcoxon rank-sum test revealed that the low-risk group had a lower predicted half maximal inhibitory concentration (IC50) of sorafenib, vincristine and methotrexate, whereas the high-risk group had lower predicted IC50 of gemcitabine, epirubicin and etoposide (Fig. 6B), which meant that this signature could be a promising indicator for chemosensitivity prediction.

### Hub lncRNA screening identified TDRKH-AS1 as a key lncRNA in HCC

**Fig. 7:**
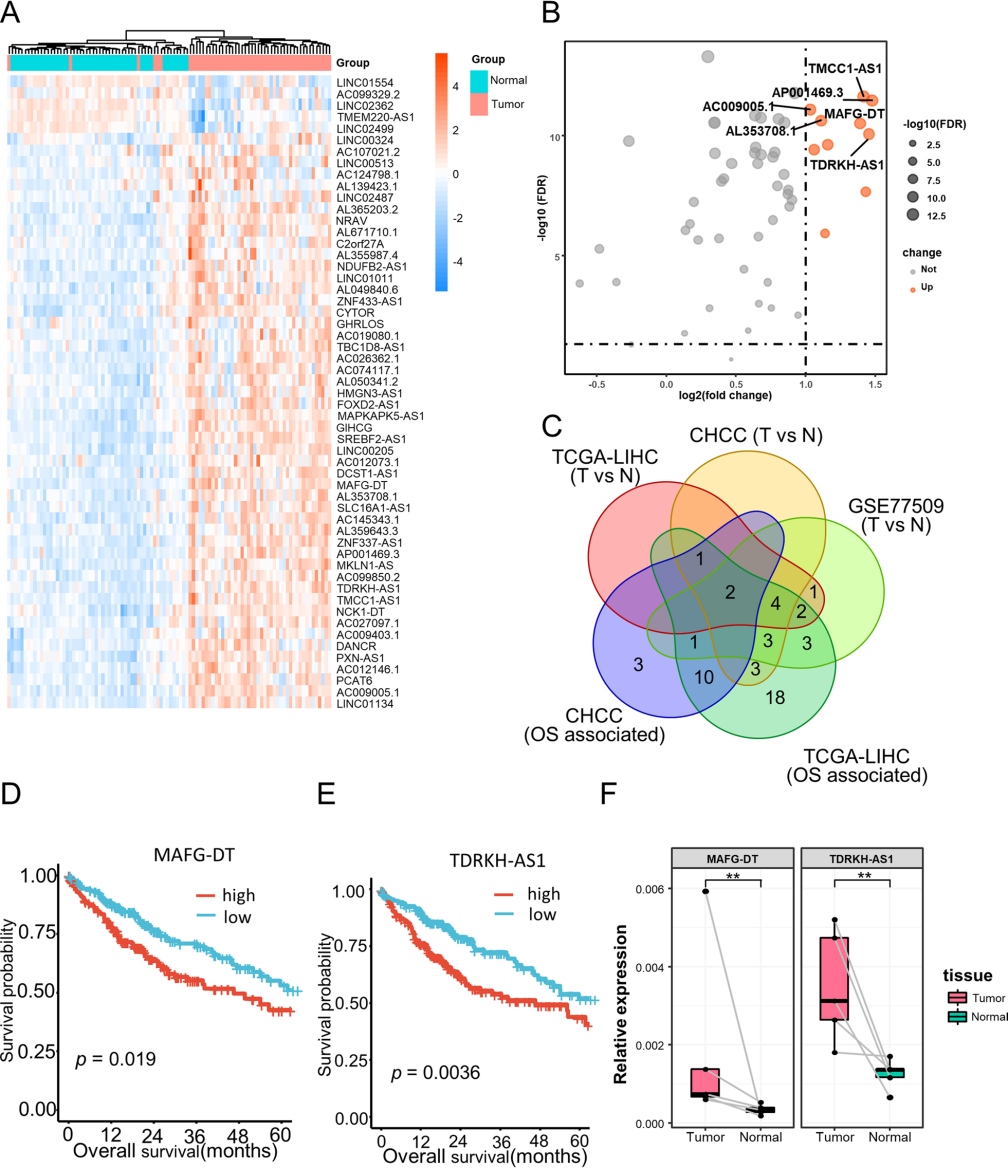
Hub lncRNA screening identified TDRKH-AS1 as a key lncRNA in HCC. Expression heatmap of the 55 lncRNAs included in the 50-LPS in the 50 paired tumor and normal liver tissues of TCGA-LIHC cohort (**A**). Volcano plot of the differential test and the top 6 differentially expressed lncRNAs with log_2_FC > 1 and FDR < 0.05 were marked in orange (**B**). Venn diagram identified two lncRNAs (TDRKH-AS1 and MAFG-DT) that were differentially expressed in TCGA-LIHC, CHCC and GSE77509, as well as associated with overall survival in TCGA-LIHC and CHCC cohort (**C**). The Kaplan–Meier curves of overall survival for TDRKH-AS1 and MAFG-DT in TCGA-LIHC (**D**, **E**). Real-time PCR validated the expression difference of TDRKH-AS1 and MAFG-DT in five paired HCC and normal liver tissues (**F**). *p < 0.05, **p < 0.01, ***p < 0.001. T, tumor; N, normal

To screen out the hub lncRNAs included in the 50-LPS, we performed differential expression analysis as well as survival analysis in multiple HCC cohorts. In the 50 tumor samples with paired normal liver tissue from TCGA-LIHC cohort, we found that most of the lncRNAs included in the 50-LPS were upregulated in tumor compared with normal liver (Fig. 7A). Especially, ten lncRNAs were significantly upregulated (log_2_FC > 1, FDR < 0.05, Fig. 7B), implying the potential function role of these lncRNAs in the carcinogenesis of HCC. Next, we further tested the expression pattern of these ten lncRNAs in CHCC cohort and GSE77509, which included 159 HCC samples and 20 HCC samples with paired normal liver tissue, respectively. Moreover, the prognostic significance of them was also evaluated in TCGA-LIHC and CHCC cohort.

As shown in Fig. 7C–E, two lncRNAs, TDRKH-AS1 and MAFG-DT, were consistently upregulated in three cohorts and associated with poor survival in TCGA-LIHC and CHCC cohort. We further validated the expression pattern of the two lncRNAs in 5 paired HCC and normal liver tissues from our center by using quantitative real-time PCR. As expected, TDRKH-AS1 and MAFG-DT were both highly expressed in HCC than normal liver (*p* < 0.01, Fig. 7F). However, TDRKH-AS1 was more abundant in relative expression than MAFG-DT, indicating a more essential role of TDRKH-AS1 in HCC tumor biology. Therefore, TDRKH-AS1 was considered as the hub lncRNA for further function investigation.

### Validation of the biological function of TDRKH-AS1 in HCC

**Fig. 8:**
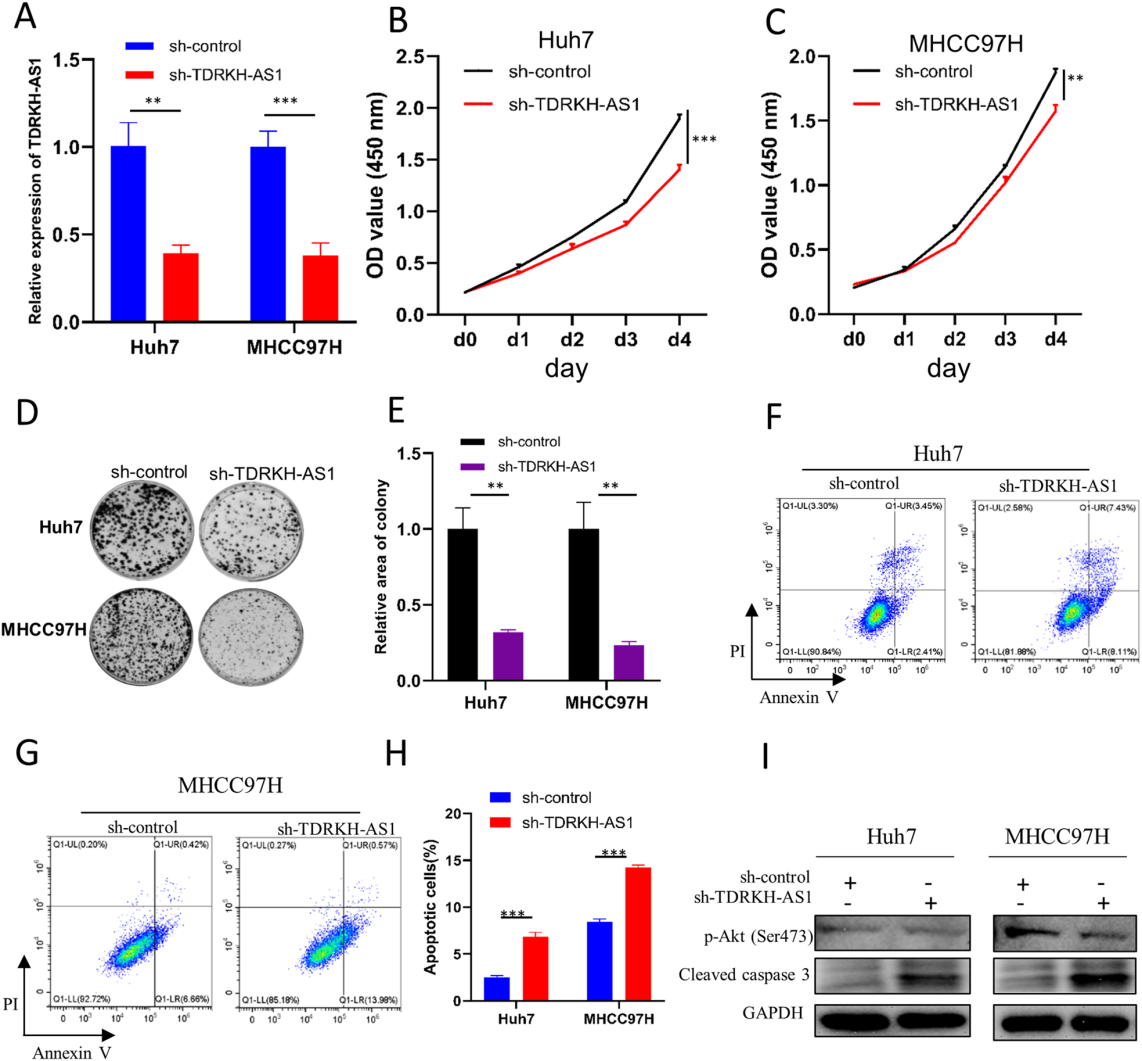
Validation of the biological function of TDRKH-AS1 in HCC. TDRKH-AS1 was successfully knocked down in Huh7 and MHCC97H cells (**A**). CCK8 assay in Huh7 (**B**) and MHCC97H (**C**) after knockdown of TDRKH-AS1. Colony formation assay in Huh7 and MHCC97H after knockdown of TDRKH-AS1 (**D**, **E**). Apoptosis assay in Huh7 and MHCC97H after knockdown of TDRKH-AS1 (**F**–**H**). Cleaved caspase3 and phospho-Akt (Ser473) were detected in Huh7 and MHCC97H by western blotting after knockdown of TDRKH-AS1 (**I**). *p < 0.05, **p < 0.01, ***p < 0.001

To validate the biological function of TDRKH-AS1 in HCC, we performed loss-of-function assays in HCC cell lines, MHCC97H and Huh7. After knocking down the expression of TDRKH-AS1 using short hairpin RNAs (shRNA) (*p* < 0.01 in Huh7, *p* < 0.001 in MHCC97H, Fig. 8A), we conducted CCK8 and colony formation assays to detect the influence of TDRKH-AS1 on cell growth of HCC in vitro. As shown in the CCK8 assay (*p* < 0.001 in Huh7, *p* < 0.01 in MHCC97H, Fig. 8B, C), the cell growth rate of both cell lines was significantly decreased after knocking down TDRKH-AS1. Restoration of TDRKH-AS1 expression in Huh7 cell line could restore cell growth (*p*<0.001, Additional file 1: Fig. S1 A, B). Similarly, the result of colony formation assay revealed that the proliferation ability of both cell lines was significantly hampered when TDRKH-AS1 was knocked down (*p* < 0.01 in Huh7, *p* < 0.01 in MHCC97H, Fig. 8D, E). In addition, the percentage of apoptotic cells were remarkably increased (*p* < 0.001 in Huh7, *p* < 0.001 in MHCC97H, Fig. 8F, G, H), and the pro-apoptotic protein, cleaved caspase3, was increased after knockdown of TDRKH-AS1 (Fig. 8I). Moreover, we found that the level of Akt phosphorylation on Ser 473 was decreased (Fig. 8I).  These results suggested that TDRKH-AS1 might be essential for tumor growth and cell survival of HCC.

## Discussion

In this study, we developed a novel individualized lncRNA signature, 50-LPS, to predict the risk of prognosis in patients with hepatocellular carcinoma after surgery. This signature was successfully validated in two external datasets and displayed high stability and robust predictive ability in all three datasets. Moreover, it was more reliable than serum AFP level, tumor vascular invasion, cirrhosis and TNM stage for OS prediction. Our study provided a novel prognostic indicator for clinical application.

As a qualitative signature, the 50-LPS exhibited a better feasibility than quantitative risk score that mostly reported. Since it was designed by using the majority voting rule based on the REOs trait of lncRNA pairs within a single sample, it did not require pre-collection of massive samples for data normalization and cut-off determination. When using the 50-LPS, the relative expression rank of lncRNAs within a single sample can be obtained by RNA sequencing, microarray or mere quantitative polymerase chain reaction, without considering sequencing depth or batch influence. Therefore, the 50-LPS can be easily applied personally. Ao et al. [35] reported a qualitative prognostic signature consisting of 20 gene-pairs for HCC and proved that qualitative signatures were reliable in prognosis prediction. However, Ao’s signature only focused on the patients with early-stage HCC and protein-coding genes. Our study revealed that qualitative signatures derived from lncRNA pairs were also powerful and feasible in prognosis prediction for HCC in early stage as well as advanced stage. A target panel will be designed for clinical translational research in our next work.

Intriguingly, the high- and low-risk groups identified by the 50-LPS was, to some extent, in agreement with previously reported molecular subtypes [9] of HCC despite of different methods and criteria used. It supports the notion that HCC patients from the two risk groups stratified by the 50-LPS might represent distinct disease entities. We observed that HCC patients in the high-risk group were biologically deficient in p53 pathway and active in E2F pathway and EMT pathway. Clinically, patients in the high-risk group exhibited more aggressive traits with high AFP level, poor tumor differentiation, vascular invasion, advanced cancer stages, and poor prognosis. As a contrast, the low-risk subclass showed retained hepatocyte-like phenotype, with moderate-high tumor differentiation, and better prognosis. In addition, nearly half of the high-risk patients had TP53 mutation. TP53 mutation could cause genomic instability and uncontrolled cell proliferation [36, 37], which might partially account for the poor OS outcome of the high-risk group. However, there is still a variation in the proportion of each risk group from different cohorts, which might result from the regional etiology difference or genetic background difference.

Although immunotherapy offers new promise to patients with HCC [38], there is still a lack of validated biological markers for predicting the therapy efficacy and guiding clinical decision-making [39]. In our study, tumor tissues in the low-risk group were higher in CD8^+^ T cell infiltration than the high-risk group, indicating that the tumor immune microenvironment in the low-risk tumor was in a more activated status. Patients from the low-risk group might be more likely to benefit from the therapy of immune checkpoint inhibitors (ICIs), yet it needs future validation. In addition, the submap results prompted that HCC patients in the low-risk group might be more likely to respond to TACE therapy, which is the standard treatment for intermediate-stage HCC [40]. Apart from that, we found that the 50-LPS could predict the efficacy of several common anticancer drugs, including sorafenib, vincristine, methotrexate, epirubicin, etoposide and gemcitabine. Taken together, the 50-LPS may be used as a tool together with other indicators in evaluating the efficacy of ICIs, TACE and some anticancer drugs, which may help improve the dilemmas in the precision treatment of HCC.

Our study has identified tens of OS-related lncRNAs in HCC and demonstrated in vitro that TDRKH-AS1 could influence the cell growth of HCC. A previous study reported that lncRNA TDRKH-AS1 could target β-catenin in the Wnt signaling pathway to promote colorectal cancer cell proliferation and invasion [41]. However, our study revealed that knockdown of TDRKH-AS1 might influence cell proliferation by inducing apoptosis in HCC. Our future researches will focus on the molecular signal pathway that TDRKH-AS1 may trigger in HCC, which would help us understand the intrinsic mechanism determining different prognosis of HCC patients more comprehensively.

Certainly, there are several limitations in our study that need to be improved. A major limitation is that the training dataset and validation datasets are all from public databases. A prospective study will be needed to validate the effectiveness and utility of the 50-LPS. Additionally, in order to improve the accuracy of OS prediction, the 50-LPS could be used in combination with other clinicopathologic features, such as AFP, VI and pathological stage in the future.

## Conclusions

In summary, current study developed a novel individualized lncRNA signature, 50-LPS, that could not only predict the prognosis of HCC patients robustly but also provide theoretical basis for precision therapy. Additionally, TDRKH-AS1 was identified as a key lncRNA in the proliferation of HCC. The 50-LPS might help guide personalized therapy for HCC patients in clinical practice.

## Supplementary Information

**Additional file 1: Table S1. **Description of the datasets analyzed in this study. **Table S2.** Sequences of primers and short hairpin RNAs. **Fig. S1. **Restoration of TDRKH-AS1 in Huh7 cells after TDRKH-AS1 knockdown. TDRKH-AS1 was successfully restored after it was knocked down in Huh7 cells**(A)**. Restoration of TDRKH-AS1 could restore the cell growth of Huh7 cells**(B)**.

## Data Availability

TCGA-LIHC can be downloaded from UCSC Xeno (https://xena.ucsc.edu/public). CHCC cohort can be accessed from NODE (https://www.biosino.org/node, ID# OEP000321). LIRI samples included in the PCAWG project can be downloaded from ICGC (https://dcc.icgc.org/releases/PCAWG). GSE77509 and GSE104580 are accessible in Gene Expression Omnibus (https://www.ncbi.nlm.nih.gov/geo).

## Abbreviations

HCC: Hepatocellular carcinoma
OS: Overall survival
lncRNA: Long non-coding RNA
50-LPS: 50 LncRNA pairs
REOs: Relative expression orderings
TCGA: The cancer genome atlas
GEO: Gene expression omnibus
PCAWG: Pan-cancer analysis of whole genomes
TNM: Tumor node metastasis
BCLC: Barcelona clinic liver cancer
CHCC: Chinese hepatocellular carcinoma
LIRI: Liver cancer RIKEN
ICGC: International cancer genome consortium
LIHC: Liver hepatocellular carcinoma
FPKM: Fragment per kilobase million
TPM: Transcripts per million
DCA: Decision curve analysis
ROC: Receiver operating characteristic curve
AUC: Area under curve
CI: Confidence interval
FDR: False discovery rate
EMT: Epithelial mesenchymal transition
GSEA: Gene get enrichment analysis
VI: Vascular invasion
TACE: Transarterial chemoembolization
IC50: Half maximal inhibitory concentration
ICIs: Immune checkpoint inhibitors
AFP: Alpha-fetoprotein
