# Supplementary material for: A novel qualitative signature based on lncRNA pairs for prognosis prediction in hepatocellular carcinoma
Source: Cancer Cell Int. 2022 Feb 22;22:95. doi: 10.1186/s12935-022-02507-z (PMC8862507; doi:10.1186/s12935-022-02507-z)
Supplement: Supplementary file 1 — Additional file 1: Table S1. Description of the datasets analyzed in this study. Table S2. Sequences of primers and short hairpin RNAs. Fig. S1. Restoration of TDRKH-AS1 in Huh7 cells after TDRKH-AS1 knockdown. TDRKH-AS1 was successfully restored after it was knocked down in Huh7 cells(A). Restoration of TDRKH-AS1 could restore the cell growth of Huh7 cells(B). [file 12935_2022_2507_MOESM1_ESM.docx]

|  | TCGA-LIHC (training) | CHCC (validation1) | LIRI (validation2) |
| --- | --- | --- | --- |
| Characteristic | *N*= 365 | *N* = 159 | *N* = 42 |
| Median age | 61 (52, 69) | 54 (46, 62) | 66 (57, 74) |
| Gender |  |  |  |
| female | 119 (33%) | 31 (19%) | 7 (17%) |
| male | 246 (67%) | 128 (81%) | 35 (83%) |
| AFP (ng/ml) | 15 (4, 265) | 115 (8, 2,524) |  |
| NA^1^ | 89 |  |  |
| Hepatitis |  |  |  |
| HBV | 58 (16%) | 159 (100%) |  |
| HCV | 20 (5.5%) |  |  |
| HBV+HCV | 82 (22.4%) |  |  |
| None | 2 (0.5%) |  |  |
| NA | 203(55.6) |  |  |
| Cirrhosis |  |  |  |
| No | 132 (36%) | 47 (30%) |  |
| Yes | 77 (21%) | 112 (70%) |  |
| NA | 156 (43%) |  |  |
| Vascular invasion |  |  |  |
| NA | 54 (15%) |  |  |
| No | 205 (56%) | 122 (77%) |  |
| Yes | 106 (29%) | 37 (23%) |  |
| TNM |  |  |  |
| I+II | 254 (74%) | 105 (66%) | 22 (52%) |
| III+IV | 87 (26%) | 54 (34%) | 20 (48%) |
| NA | 24 |  |  |
| NA, not available | | | |

**Table S1 Description of the datasets analyzed in this study**

**Table S2 Sequences of primers and short hairpin RNAs**

| Name | Sequence (5’-3’) | |
| --- | --- | --- |
| TDRKH-AS1 | forward | GAAACAGAACATTGCTAGTGCCC |
|  | reverse | GAGTCAGAAGAGTGGTTGTCCTTG |
| MAFG-DT | forward | AGAACCGCGAAAGGCTACTG |
|  | reverse | TAAAGCCGGTCGTGGAGATG |
| β-actin | forward  reverse | CATGTACGTTGCTATCCAGGC  CTCCTTAATGTCACGCACGAT |
| sh-TDRKH-AS1 |  | CCTCTCAGTCACTGCTCTT |
| sh-control |  | CCTAAGGTTAAGTCGCCCTCG |

**Fig. S1 Restoration of TDRKH-AS1 in Huh7 cells after TDRKH-AS1 knockdown**


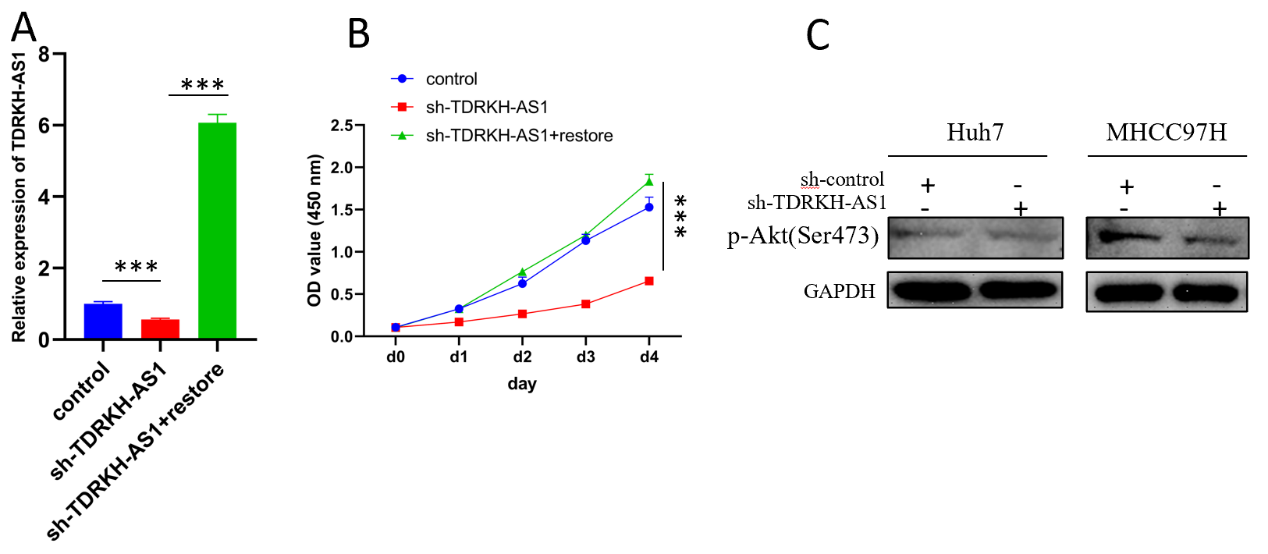


TDRKH-AS1 was successfully restored after it was knocked down in Huh7 cells**(A)**. Restoration of TDRKH-AS1 could restore the cell growth of Huh7 cells**(B)**.
